# Effect of Wood Biomass Ash Storage on the Properties of Cement Composites

**DOI:** 10.3390/ma14071632

**Published:** 2021-03-26

**Authors:** Ivana Carević, Nina Štirmer, Marijana Serdar, Neven Ukrainczyk

**Affiliations:** 1 Department of Materials, Faculty of Civil Engineering, University of Zagreb, Fra Andrije Kačića Miošića 26, 10000 Zagreb, Croatia; nina.stirmer@grad.unizg.hr (N.Š.); marijana.serdar@grad.unizg.hr (M.S.); 2 Institute of Construction and Building Materials, Technical University of Darmstadt, Franziska-Braun-Straße 3, 64287 Darmstadt, Germany; ukrainczyk@wib.tu-darmstadt.de

**Keywords:** wood biomass ash, cement-based materials, cement partial replacement, aging, hydration, carbonation

## Abstract

Since ash from wood biomass mostly ends up in landfills, recent research has focused on finding its economic and environmental added value as a potential new raw material in the construction industry. However, for wood ash to be used on an industrial scale in construction, a strategy for its proper storage must be defined. Proper storage of WBA is important to ensure quality control for applications in cementitious composites. This work investigated the aging of wood biomass ash (WBA) collected from five different power plants in Croatia and its influence on the performance of cementitious composites. WBA and cement pastes were investigated at different aging times (up to one year) using thermogravimetric analysis (TGA), powder X-ray diffraction (XRD), isothermal calorimetry and initial and final setting times. The results showed that storage of WBA in closed and open containers resulted in carbonation and hydration of mainly free lime and periclase, respectively, which affected the reactivity and setting times of WBA cement pastes.

## 1. Introduction

**Figure 1:**
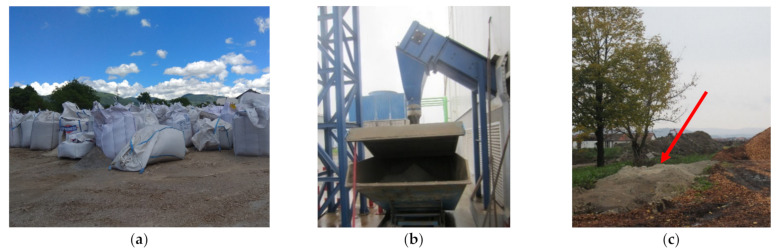
Different approaches to wood biomass ash (WBA) storage: (**a**) in plastic bags; (**b**) in the container; (**c**) landfilling.

The use of wood biomass as one of the renewable sources of energy has increased significantly in recent years [1,2]. This results in a growing amounts of wood biomass ash (WBA) waste being generated in such energy power plants. Statistical European data show increased use of solid biofuels [3], with solid biomass having one of the largest increases [4]. The authors estimated that approximately 15.5 t × 10^6^ t of WBA [5] was produced in 2015, while the projection of WBA produced is expected to triple by 2020, implying the need for a future strategy for WBA management. WBA is mostly disposed [5,6], so recent research has focused on finding added economic and environmental values for WBA, e.g., as a potential new raw material in the construction industry [7,8,9,10,11]. The recovery and reuse of WBA poses logistical challenges to owners and managers of wood biomass power plants, as well as companies that collect WBA from power plants, as shown in Figure 1. In particular, the storage pre-treatment and transportation from the power plant to the user of this by-product seems to be a critical point. Proper storage of WBA should not only include prevention of pollution and healthy concerns but may also significantly affect the quality of the WBA. Therefore, detailed characterization of WBA is the first and most important step to decide about the appropriate treatment and use of WBA [12,13]. Namely, for the application of WBA as a mineral additive in cementitious composites, there is a need to understand the aging of WBA in terms of chemical and mineral changes induced during the various storage conditions.

WBA has hydraulic and latent hydraulic (pozzolanic) properties and therefore has shown great potential to partly replace conventional cements and thus improve environmental impact of construction materials. Cementitious raw materials in general and thus also WBAs are highly hydrophilic, i.e., they readily absorb water vapor, which is commonly referred to as pre-hydration [14]. Pre-hydration leads to partial hydration of the surface of the cementitious grains. This phenomenon can occur during the production process, e.g., already in a clinker mill, or later during storage [15]. Based on the laboratory tests conducted by [16], cement loses its fineness during prolonged storage. When the cement absorbs moisture, the hydration process begins and forms chemical hydration bonds between the aggregated cement particles, also called “lumps”. Initially, the formed lumps may break and become fine, but when the storage time is extended, the lumps cannot break and therefore become rigid. As cement ages, its reactivity decreases, resulting in decreased compressive strength, prolonged setting time, and increased water demand [17,18,19]. It has been found that cement aging leads to the formation of early reaction products, mainly ettringite and syngenite, on the surfaces of clinker grains [20], and carbonates. Therefore, the optimal shelf life of Portland cement is not more than 3 months. If it is stored for more than 3 months, the properties (e.g., strength) of the cement should be tested to determine whether aging had significantly affected its properties. However, if properly stored under dry conditions and sufficiently low relative humidity (20 °C and <35% r.h.), cement can be expected not to change its properties for several years [21].

Like in use of cement, also when considering the use of wood biomass ash (WBA) as a supplementary cementitious material in the concrete industry, it is important to understand the aging, i.e., the quality change during storage of WBA. There are studies on the process of natural [22,23,24,25] and accelerated aging of materials [26], which are also relevant in the case of WBA [27,28]. It had already been established that pre-hydration and carbonation can have a positive effect on the application of WBA in agriculture and forestry by lowering its pH value [28]. In addition, pre-hydration may have a positive effect on reducing dust generation during transport and application of WBA, as well as reducing Ca leaching [26,27,29,30]. WBA has a high content of CaO (and MgO), which can initially be in the form of free lime (and periclase) and can spontaneously hydrate and rapidly carbonate under humid conditions [31]. In particular, the amount of free CaO against carbonate products depends not only on the type of a wood biomass being burned but also on the firing, storage and transport conditions of the WBA. Any change in the chemical/mineralogical composition of WBA during storage could have an impact on the properties of composites made with WBA. For this reason, the storage of WBA becomes a challenge: it must be ensured that WBA does not lose its reactivity during storage.

The main objective of this work was to evaluate the influence of WBA aging on the performance of cement composites, as a step towards defining an optimal strategy for WBA storage when used as a raw material in the concrete industry. The research focussed on five different types of WBA samples collected from power plants using grate combustion system (which are not water-cooled). WBAs collected from such plants show self-cementing properties over time [31,32,33,34] and therefore more knowledge about their use and storage is required. 

## 2. Materials and Methods

### 2.1. Materials

**Figure 2:**
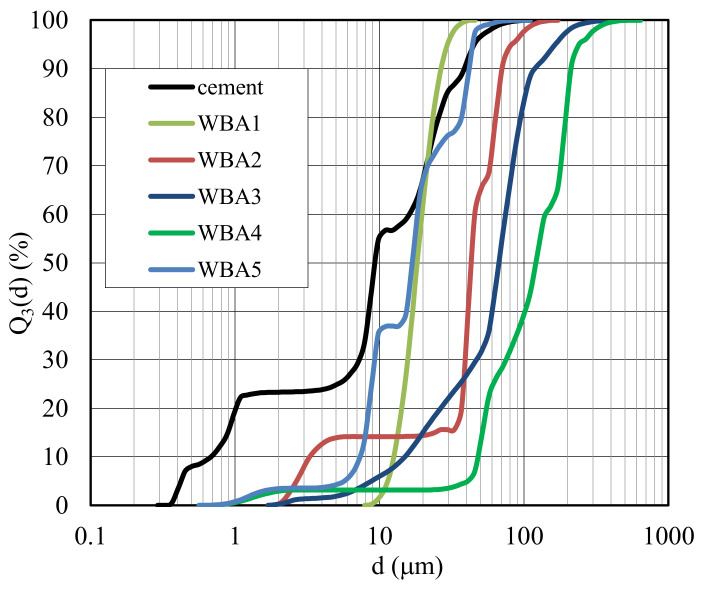
Particle size distribution [7,36].

Wood biomass fly ashes from five different power plants in Croatia were used in this study. The characterization of the used WBAs and cement (chemical composition, particle size distribution, particle morphology) has already been described in detail by [7]. A summary of the chemical and physical properties of WBAs and cement is presented in Table 1 and Figure 2. The input parameters of the power plants (average combustion temperatures, technology of combustion, and biomass used in the power plants) are given in Table 1 and are discussed in detail in [35].

WBAs were collected from October to January and periodically tested by thermogravimetric measurements, while chemical and physical properties of WBAs were done from January to April, 2018. During the WBA collection, an attempt was made to take “fresh” WBAs that had just come out of the furnace or chimney (“fresh” top WBA from inside the container (WBA1, WBA2, WBA4, WBA5) or plastic bag (WBA3) at the power plant). Cement paste was prepared in May/June 2018 and in the same period after one year of WBA aging. Powder X-ray diffraction (XRD) and calorimetric measurements, as well as the properties of the pastes in the fresh state, were measured on WBAs stored in plastic bags kept in closed plastic containers under laboratory conditions (20 °C and 50% RH). About 5 kg of WBA were collected at power plant, of which 100 g were used for TG and XRD measurements at different storage conditions (one representative sample per different WBA was used for test). The other part of the collected WBA was used for paste preparation, using fresh WBA (in initial state) and after one year.

Two storage conditions were simulated (one sample per condition): (i) in closed containers to simulate storage in concrete plants, and (ii) under open laboratory conditions to simulate outdoor storage in power plants. TG analysis of WBAs and cement were performed at different sample ages: Samples were tested immediately after collecting of WBA (initial condition) and after 7 and 28 days and 1 year of storage in closed plastic bags—simulating storage in closed containers. Part of the samples were stored in open containers exposed to the air and humidity conditions of the laboratory (20 °C and 50% RH)—simulating open landfilling. These samples were tested after 3 and 6 months of WBA age of the samples.

### 2.2. Thermogravimetric Measurement

Thermogravimetric (TG) measurements of WBA and cement stability were performed on a LECO TGA 701 (LECO Corporation, MI, USA) using a ceramic vessel filled with approximately 1 g of powder sample. For all samples, the temperature interval of the measurement ranged from 35 to 950 °C at a heating rate of 20 °C/min. The sample chamber was filled with inert gas (nitrogen, flow: 30 mL/min) to prevent oxidation during the measurement. Before measurement, the samples were dried for at 35 °C. for 15 min to lose moisture. Calcite (CaCO_3_) values were determined from the TG measurement. Calcite decomposes to CaO and CO_2_ at temperatures above 600 °C. Mass loss (*WL_CaCO3_*) can be used to determine calcite using the molecular weight of CaCO_3_ (*m*_CaCO3_ = 100 g/mol) and CO_2_ (44 g/mol) as follows:

CaCO_3_*= WL*_CaCO3_*× m*_CaCO3_*/m*_CO2_ (1)

The mass loss of the sample as delivered from 35 to 950 °C can be defined as a baseline from which incremental prehydration of the sample powder can be defined as additional mass loss in the same temperature range of that powder at a later time. Therefore, the prehydration index (PI) can be calculated as follows [37]:

(2) $$\mathrm{PI}=\left[\left(\frac{\Delta m}{m_{i}}\right)-\left(\frac{\Delta m_{0}}{m_{0}}\right)\right]\times 100$$

where:

- ∆*m*—mass loss between 35 to 950 °C, or mass loss between 35 to 600 °C;
- ∆*m*_0_—mass loss between 35 to 950 °C or mass loss between 35 to 600 °C for sample in the initial state;
- *m*_i_—mass of sample at 950 °C or mass at 600 °C;
- *m*_0_—mass of sample at 950 °C or mass at 600 °C in initial state.

PI_950_ includes carbonation from a direct effect of ageing and a secondary effect of reaction with water. Within PI, carbonation includes the formation of CaCO_3_ by the direct carbonation of free lime(CaO) in the sample, the carbonation of Ca(OH)_2_ formed by the hydration of CaO, and the reaction of free lime with water vapour water in the air, forming silica and portlandite phases [37]. To separate the influence of hydration and carbonation, PI_600_ was also investigated as mass loss between 35 and 600 °C compared to the initial state.

### 2.3. XRD Analysis 

Diffractograms of the samples were obtained by powder X-ray diffraction (XRD) using a Philips MPD 1880 diffractometer (Philips, Almelo, The Netherlands). Powdered samples were scanned between 5° and 70° with a step size of 0.02° using CuKα_1,2_ radiation. Quantification of free CaO and free MgO minerals was analyzed using a Bruker desktop diffractometer (Bruker, Ettlingen, Germany) with a fast linear LYNXEYE detector (5 degrees opening). A detailed description of the method can be found in [7]: The quantitative analysis was based on the adiabatic principle with auto flushing [38] where the relationship between the peak area of the characteristic X-ray reflection Ii is directly proportional to the weight fraction of the component by the factor ki, which contains the mass absorption coefficient of the total sample. The ki values were determined by mixing pure phases (CaO and MgO) with corundum (Al_2_O_3_) in a 50:50 weigh ratio. Each sample prepared for QXRD (1 g) was mixed with a fixed amount of corundum (0.1 g) and then ground and homogenized in an agate mortar under acetone. Appropriate corrections for peak overlap were meticulously applied by inference to the (measured) intensities of the pattern due to the pure phases.

### 2.4. Isothermal Calorimetry 

Heat release data of cementitious pastes were obtained by isothermal calorimetry, where the water to cementitious materials ratio was 0.5 and 15% of OPC was replaced by wood biomass ash. About 20 g of the paste samples were mixed for 3 min, and 6 g of the sample was placed in a sealed ampoule and lowered into the calorimeter (TA Instruments, New Castle, DE, USA), which had been conditioned to 20 ± 0.05 °C. The WBA used in this test was over one year old. 

### 2.5. Mix Design

CEM I 42.5 R cement and water at a temperature of 20 ± 2 °C were used to prepare paste samples. The reference mix (P0) was prepared using 500 g of CEM I 42.5 R and 125 g of water according to EN 196-3 [39], while 15% WBA was used as cement replacement for the preparation of the other pastes. Based on previous research [36,40] and due to workability issues, 15% of WBA was used as cement replacement in this experiment. The amount of water in the paste samples was varied to obtain standard consistency. The standard consistency of the paste was determined using the Vicat apparatus according to the standard EN 196-3 [39] where the Vicat must penetrate to a point 6 ± 2 mm from the bottom of the Vicat mould. In addition to standard consistency, temperature and setting time were also tested.

## 3. Results and Discussion

### 3.1. Aging of WBA Samples Stored in Closed Containers

**Figure 3:**
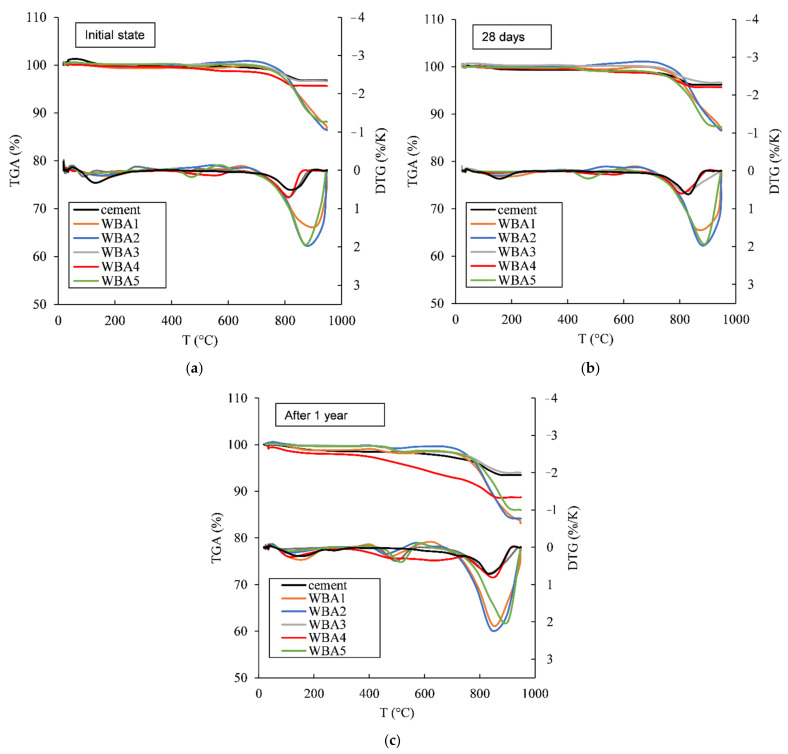
Thermogravimetric (TG) and differential thermogravimetry (DTG) curves of cement and wood biomass ash (WBA) samples for: (**a**) initial state; (**b**) 28 days, and (**c**) after 1 year (the data are available in Supplementary Materials).

To investigate shelf life and changes in properties of WBA in closed environment, the changes in mineralogical composition were monitored by TG and XRD for one month and after one year. Figure 3 shows the TGA of WBA powders and cement measured immediately after collecting WBA powders from the power plants and after one year of storage in the closed plastic containers. From the TGA diagram, usually three main peaks can be distinguished for prehydrated cement [37,41,42]: The first peak between 50 and 250 °C is attributed to the decomposition of hydration products (C-S-H, ettringite and monosulphate); the second peak refers to the decomposition of portlandite between 400 and 600 °C to CaO and H_2_O, and the third peak at about 700 °C is attributed to the decomposition of calcium carbonate to CaO and CO_2_. All WBA samples showed a distinctive third peak in the initial state (immediately after collection) or a large share of CaCO_3_ immediately after collection (Figure 3a). The CaCO_3_ content of the WBA samples immediately after collection is in the following order from higher to lower values: WBA2 (32.74%) > WBA1 (28.72%) > WBA5 (27.26%) > WBA3 (8.04%) > WBA4 (6.99%). In this research, WBA samples with higher CaCO_3_ values after collection were mainly found to be primarily samples that also had finer particles and a higher proportion of free lime. The authors stated that the temperature in the grate combustion can reach up to 1000–1200 °C, in fluidized bed combustion plants combustion temperatures are lower (less than 900 °C), while in the pulverized fuel combustor plants temperatures can reach up to 1600 °C compared to other combustion systems. The mineralogy of WBA changes with the temperature of furnace: At lower temperatures (around 600 °C), carbonates are the main phases, while at higher temperatures (up to 1300 °C) free MgO and CaO are expected to be main phases [43]. According to Table 1, all temperatures in the power plants where WBA was collected were lower (700–950 °C), but these temperatures are average values and higher temperatures in the furnace could be expected as well. Samples WBA5 from power plants with bubbling fluidized bed and WBA samples WBA1 and WBA2 from power plants with pulverized fuel combustors showed higher values of CaCO_3_ content compared to samples collected from power plants with grate combustion technology (WBA3 and WBA4).

**Figure 4:**
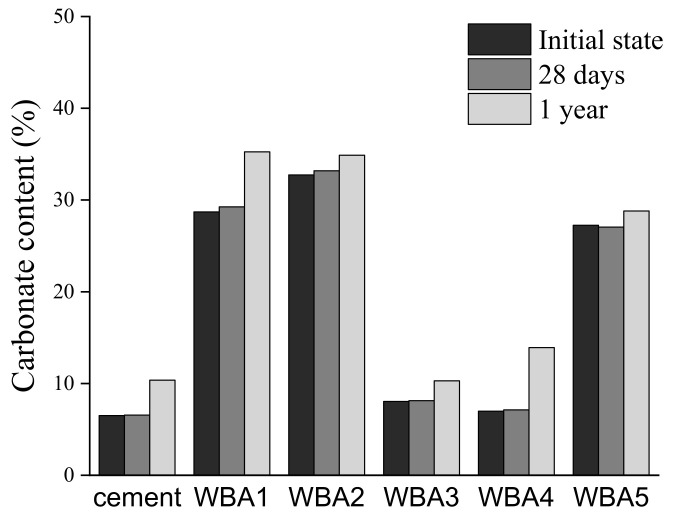
Carbonate content for WBA samples and cement per time according to equation 1.

There were no significant changes in the TG and DTG curves after 28 days (Figure 3b), but after one year of aging in the closed containers, it is evident from the TGA (Figure 3c) that there is an increase in the amount of hydration products (first and second peak) and carbonation products (third peak). It is necessary to additionally highlight the results for the WBA4 sample after one year: There is an obvious mass loss over a wide temperature range, suggesting calcium silicate hydrate [21]. This could indicate that WBA4 sampling or storage conditions might have affected the moisture condition, although the seal has been checked in several time periods. Additionally, TG measurements were repeated for this sample, but the results were the same. In any case, apart from carbonation, the aging of WBA sample indicated hydraulic and/or pozzolanic reactivity. The chemical composition of WBA4 differs from other WBAs where higher pozzolanic oxides can be seen which could affect the aging process, but this needs to be confirmed by further research. Changes in carbonate content over time for each tested sample stored in closed containers, determined according to Equation (1), are shown in Figure 4. A large amount of carbonate phase is visible in all WBA samples, especially in WBA 1, WBA 2, and WBA5. It must be emphasised that when the carbonate content is high, as in these WBA samples, it is necessary to select the correct method for determining content of unburned carbon (UC), especially LOI testing, as one of the main characterization tools for supplementary cementitious materials (SCM) [44,45,46]. Generally, LOI values of WBA from Table 1 determine WBA as low-quality ash for application as mineral admixture if compared with requirements given by EN 450-1 [47]. Mineral additions with high content of UC can lead to increase water demand due to a high specific surface area [48].

**Figure 5:**
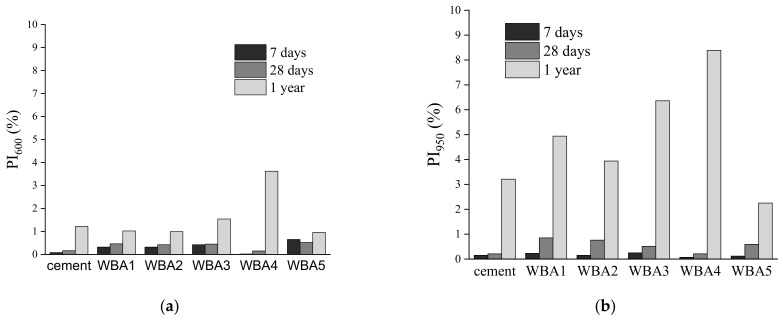
(**a**) PI_600_ value for WBA samples (7 days, 28 days, and 1 year) for temperatures between 35 and 600 °C; (**b**) PI_950_ value for WBA samples (7 days, 28 days, and 1 year) for temperatures between 35 and 950 °C.

In order to distinguish the influence of carbonation and hydration of the WBA samples, the pre-hydration index (Equation (2)), i.e., the change in mass loss of the tested sample in time per initial state, i.e., normalized per initial state) was calculated for different temperature ranges and shown in Figure 5. PI_600_ mainly includes the influence of hydration, while PI_950_ includes the carbonation and hydration of the WBA samples normalized to the WBA initial state results.

**Figure 6:**
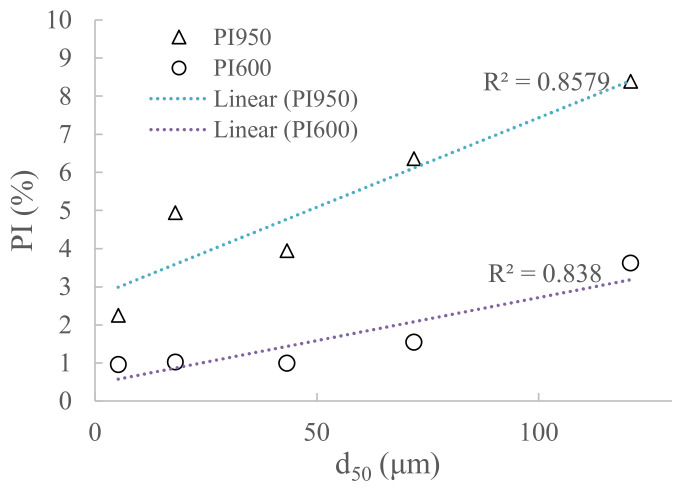
Effect of fineness of WBA samples on pre-hydration index after 1 year (PI_950_ and PI_600_).

From Figure 5, it can be seen that WBA storage in closed containers for 28 days showed no increasing PI values, or no significant change in TGA results was observed for WBA aging for 28 days. PI_600_ values after 28 days were from 0.15% (WBA4) to 0.51% (WBA5), while for PI_950_ the values were from 0.21 (WBA4) to 0.85% (WBA1). However, after one year, there was a slight increase in mass loss between 35 and 600 °C and a more significant increase in mass loss between 35 and 950 °C, suggesting that carbonation of WBA samples stored in closed containers is more pronounced during aging. Here, sample WBA4 stands out as it shows the largest mass increase of all the samples studied from the time of collection (initial state) until storage in the closed container after one year. For sample WBA4, the values for indices PI_600_ and PI_950_ were 3.62% and 8.39%, respectively. Sample WBA3 had the second highest mass increase during aging (PI_600_ and PI_950_ were 1.54% and 6.36%, respectively), while sample WBA5 had the lowest mass increase (0.96% and 2.25%). Both samples with the highest increase in carbonation products (WBA4 and WBA3) were from power plants using grate combustion technology (with higher combustion temperature), implying that aging could have a greater impact on the WBA generated from this technology, as it promotes the complete decomposition of carbonates (CaCO_3_ and dolomite stones) into the free lime (and periclase). The influence of each chemical and physical property of the WBA on the increase in carbonation products was statistically analysed and the only parameter that had a statistically significant influence was particle size (Figure 6). Samples with larger particle sizes exhibited faster carbonation. A similar trend can be seen in the study of [28] where WBA samples with larger particles size from grate combustion showed higher mass increase when stored outdoors compared to other samples. The possible influence of combustion technology on the carbonation process of WBA samples should be further investigated. 

**Figure 7:**
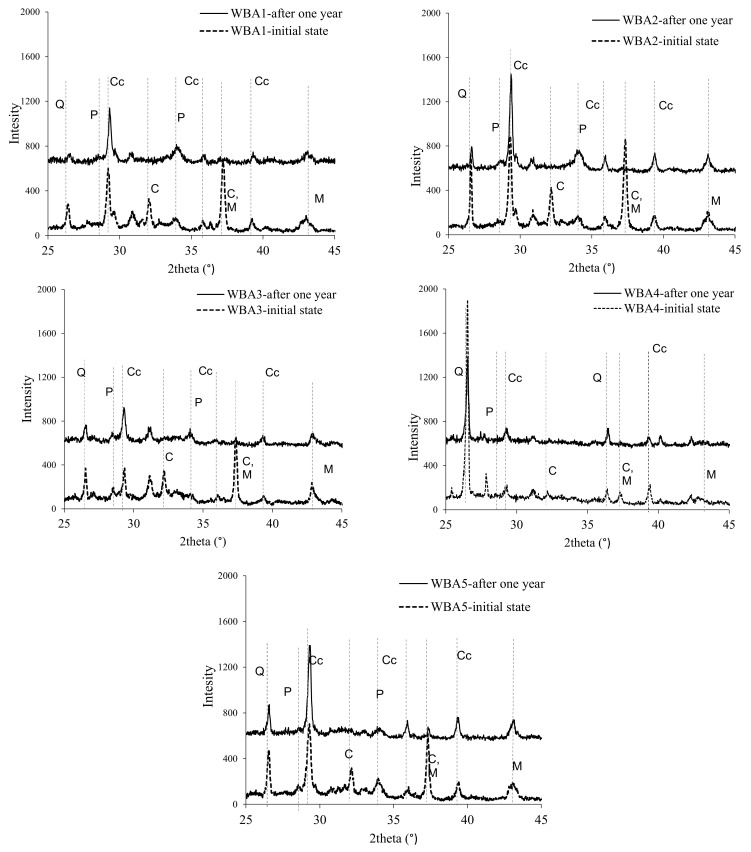
XRD diffractograms of WBA samples after collecting from power plants and after one year of storage in closed containers (the data are available in Supplementary Materials).

The results of XRD analysis of the tested WBA samples are shown in Figure 7 where the main phases were identified as follows: Cc—calcium carbonate (CaCO_3_), Q—quartz (Si_2_O), C—calcium oxide (CaO), M—periclase (MgO), and P—portlandite (Ca(OH)_2_). Comparing the diffractograms of the initial samples and those after one year of storage in closed containers, a strong decrease in the peaks of calcium oxide (lime) and magnesium oxide (periclase) can be seen. Therefore, some decrease in the amount of free CaO in closed containers can be expected. Materials with a high amount of free MgO and free CaO in concrete increase the risk of volume instability (swelling) during the hydration process, and the formation of cracks [49,50,51]. According to Table 1, the collected WBA have higher free CaO values compared to cement, but the soundness test results [36] showed that cement paste mixes with 15% WBA as cement replacement met the requirement of the standard EN 450-1 [47]. Free CaO is very sensitive to atmospheric moisture and carbon dioxide [52,53,54], leading to phase changes within the material [28,55,56]. The present results suggest when WBA is stored in closed containers, the free lime is stabilised over time, i.e., its reaction with water and CO_2_ is significantly delayed due to their limited availability by mass transport. This form of stabilization could be positive, as a decrease in free lime leads to a lower risk of volume instability when higher dosages of WBA are used as a cement replacement.

**Figure 8:**
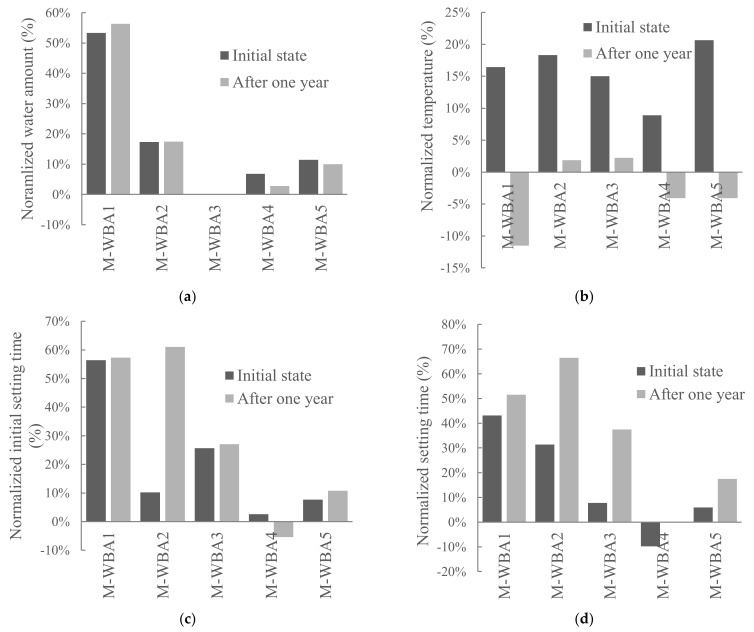
Properties of fresh paste as a function of the type and age of the WBA used as a 15% cement replacement: (**a**) Standard consistency; (**b**) Paste temperature; (**c**) Initial setting time; (**d**) Final setting time (Values are normalized to the reference mix; the data are available in Supplementary Materials).

In order to evaluate the effect of aging in closed containers on the properties of cement pastes, setting time, standard consistency as well as cement paste temperature were tested with WBA delivered and with WBA stored in closed container for one year. Figure 8 shows the results of the standard consistency, setting time, and temperature of the pastes with 15% WBA as cement replacement (M-WBA_i_). WBA was used after collection from power plants (initial state) and after one year of aging, stored in closed containers (after one year). All results were normalized with respect to the reference mix.

From Figure 8, it can be concluded that the water requirement increased at 15% cement replacement by WBA for both fresh and aged WBA. This is in agreement with [57,58]. In all samples prepared with aged WBA, the temperature of the paste was lower than samples prepared with fresh WBA. A possible explanation could be a decrease in free CaO (and periclase) after one year of storage, as shown in Figure 7. The free CaO (and periclase) hydration reaction releases heat, which can cause a temperature rise when fresh WBA is used. The initial setting time was increased for all cement paste samples, regardless of the age of the WBA used, except for sample WBA4. The same trend is seen in the pre-hydrated (aged) cement according to the research presented in [19]: The final setting time was prolonged while the initial setting time was decreased. The pre-hydrated cement showed unusual setting behaviour: Pre-hydrated cement shows an initial set before becoming plastic again and then subsequently hardening in a conventional manner, albeit much delayed [17]. As cement ages, hydration products form on the surface of its grains: products of calcium silicates and hydrates of calcium aluminate, portlandite and gypsum. The formation of gypsum disturbs the sulfate balance and delays the formation of ettringite and slows the setting time. This results in a quasi-flash set that disappears as the gypsum slowly goes into solution and reacts with the calcium aluminate hydrates [17]. Most likely, this trend is related to the WBA4 sample in terms of a different chemical composition in contrast to the other WBAs tested, which have lower CaO content and higher pozzolanic oxide content. For all the tested paste samples with aged WBA, the final setting time increased, i.e., the setting time is expected to increase with the aging of the WBA. The cement behaves in the same way with longer storage time.

**Figure 9:**
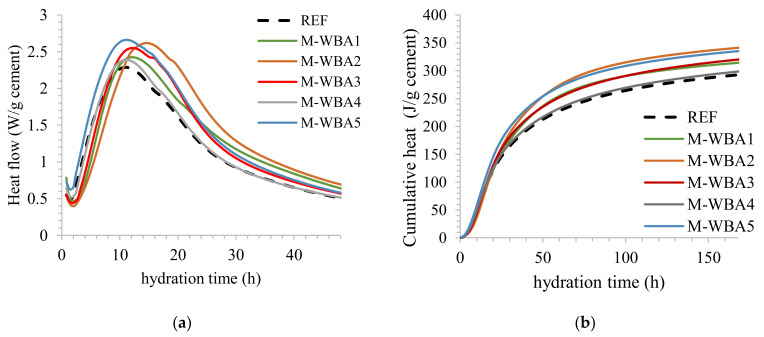
The influence of WBA after 1 year on the reactivity of pastes: (**a**) heat flow; (**b**) cumulative heat flow (the data are available in Supplementary Materials).

Finally, it has been observed in previous studies that wood ash contributed to the evolution of heat during hydration [41]. Namely, with the addition of wood ash, more heat per g of cement was released during hydration. To evaluate whether aged WBA stored in closed container had the same effect on reactivity, the heat release was monitored on samples stored in closed containers for one year. Figure 9a,b shows the influence of aged WBA after one year on the reaction rates of cement pastes using isothermal calorimetry.

The increase in heat of hydration due to the use of pozzolans is usually attributed to the filler effect, i.e., particles smaller than cement fill spaces and allow higher nucleation and growth of hydration products and thus reactivity of cement [59]. However, since the WBA particles are larger here, compared to cement, the filler effect alone cannot be used to explain results obtained. Alternatively, hydration of free lime (and periclase) and CaO- (and Al_2_O_3_-) rich phases could be a possible explanation. In general, the addition of WBA increases and delays the heat flow, with the exception of the sample WBA5. This sample has the smallest particle size of all the WBA samples tested. For all other samples, the induction period is prolonged by the addition of WBA, which is consistent with [41]. This explains the prolonged setting times of all samples, except for the sample WBA5. The cumulative heat flow of the pastes with WBA is higher compared to the reference mix as follows: WBA2 (2393.16 J/g) > WBA5 (2353.35 J/g) > WBA3 (2254.25 J/g) > WBA1 (2204.42 J/g) > WBA4 (2106.65 J/g). According to studies on pre-hydration of cement [60], increased setting time, decreased compressive strength and heat of hydration, and altered rheological properties are expected. Here it is shown that there was no significant influence on the reactivity of the pastes during WBA aging. 

**Figure 10:**
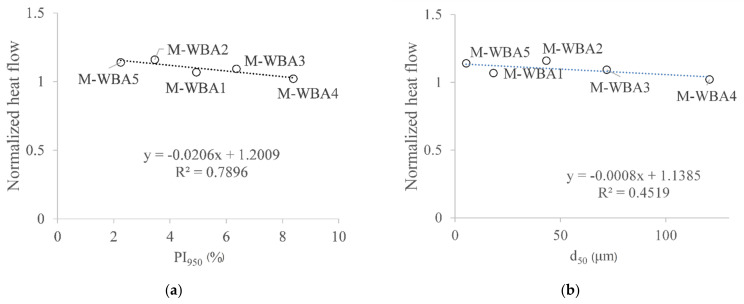
(**a**) Normalized cumulative heat flow after 168 h and PI_950_ values after one year of storage of WBA samples; (**b**) normalized cumulative heat flow after 168 h and d_50_ of WBA samples.

The cumulative heat release of pastes with 15% WBA as cement replacement was normalized to the cumulative heat release of the reference paste mixture and the influence of PI_950_ values and particle fineness is shown in Figure 10a and Figure 10b, respectively.

Figure 10a shows a reasonable linear relationship (R^2^ = 0.7896) between the pre-hydrated index of WBA samples and the normalized cumulative heat flow where with higher PI_950_ index the cumulative heat flow of the pastes decreases. Unfortunately, the influence of WBA itself during the collection was not analysed here (initial state). Therefore, the influence of WBA alone as a cement replacement and the influence of WBA aging on the reactivity of the pastes cannot be separated. Based on Figure 10b, it can be said that with smaller WBA particles, the cumulative heat flow is higher, but the linear dependence is not so convincing (R^2^ = 0.4519) compared to the previous linear relationship (between the PI index and the cumulative heat flow). Sample WBA4 has the highest influence on the reactivity of the pastes with WBA. Sample WBA4 has the highest PI value. Based on the TGA measurement (Figure 3), only WBA4 shows noticeable changes in the TG analysis after one year of sample aging, as indicated by a typical DTG peaks for the carbonated sample [61]. This WBA has the largest particles among all the WBAs tested, with half of the particles smaller than 120.7 µm. The WBA4 sample is richest in pozzolanic oxides, similar to coal fly ash [62], and one would expect an increase in heat hydration [63]. However, this is not the case here. Most likely, both parameters, the properties of the WBA and the aging of the samples used in pastes, had an influence on the reactivity and thus on the mechanical and durability properties when used in mortars and pastes. 

### 3.2. Aging of WBA Samples Stored in Opened Containers

**Figure 11:**
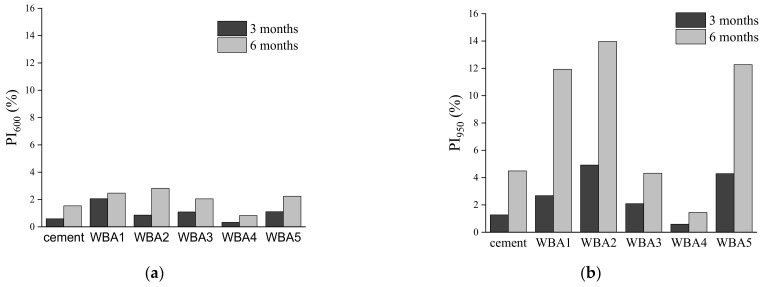
(**a**) PI_600_ value for WBA samples (aged for 3 and 6 months) for the temperature range between 35 and 600 °C; (**b**) PI_950_ value for WBA samples (aged for 3 and 6 months) for the temperature range between 35 and 950 °C.

In the previous session, it was shown that the ageing of WBA behaves similarly to that of cement when stored in a closed environment. Since in the EU about 70% of WBA is disposed of in landfills [64] and in Croatia 61% [35], it is necessary to analyse whether the storage of WBA in opened landfills (outside) has a stronger influence on WBA properties. In order to evaluate changes in mineralogical composition during outdoor storage in landfills, a TGA was performed. WBA samples were stored in open containers, which allowed reaction with moisture from the air. Using TG measurements of the WBA powders after 3 and 6 months of storage in open containers, the carbonate content determined according to Equation (1) was again compared to the initial state. The results indicate an increase in the carbonate phases from 18.6% (WBA4) to 52% (WBA5) for the samples stored in the open container. In order to distinguish the influence of carbonation and hydration of the WBA samples stored in open containers, different pre-hydration indices were calculated, Figure 11.

**Figure 12:**
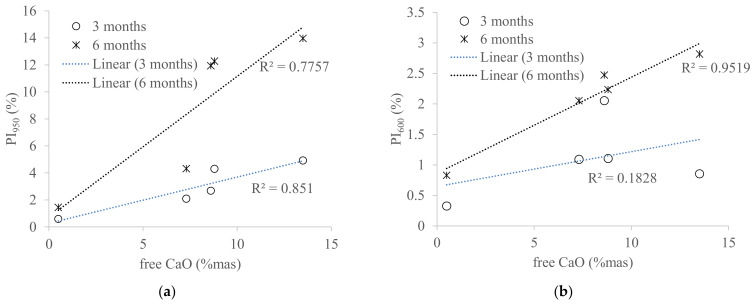
Effect of free CaO of WBA samples on prehydration index: (**a**) PI_950_ and (**b**) PI_600_.

Comparison between the two storage conditions clearly showed that WBA samples stored in air exhibited faster aging, i.e., a more significant increase in PI values in a shorter time (Figure 11) than those stored in closed containers (Figure 5). WBA stored in open containers showed an increase in PI values as follows: PI_600_ values increased from 0.83% (WBA4) to 2.82% (WBA2) after 3 months in air-open storage, while for PI_950_ they increased from 1.45 (WBA4) to 14% (WBA2). A significant increase in mass change due to hydration and carbonation is observed in the following order (from higher to lower values): WBA2 (13.97%) > WBA5 (12.27%) > WBA1 (11.93%) > WBA3 (4.31%) > WBA4 (1.45%). In the case of open storage, a different reaction trend of the phases was noticed: Those WBA samples that had a higher proportion of free CaO reacted faster by reacting with water vapor from the air (ambient humidity) and hydration occurred. The high correlation of PI_600_ and PI_950_ with free CaO (R^2^ = 0.9519 and R^2^ = 0.7757) can be clearly seen in Figure 12, indicating that in the case of open storage, carbonation is pronounced in samples with high values of free CaO. This can be seen in the TGA diagrams through the second peak indicating the decomposition of portlandite (Figure 3).

## 4. Conclusions

Wood biomass fly ashes from five different power plants were used in this study to evaluate the effect of WBA aging on the performance of cement composites. No significant change was observed in TG results after WBAs aging in closed containers for 28 days. However, after one year of storage of WBAs in closed containers, an increase in the amount of hydration and carbonation products is visible while the amount of free CaO was decreased. It was noticed that ageing has a greater impact in the case of WBA produced by grate combustion technology, but the possible influence of combustion technology on carbonation process of WBA samples should be further investigated. In general, samples with larger particle sizes exhibited faster carbonation. The results obtained indicate that when WBA is stored in closed containers, the free lime is stabilised over time, which is positive regarding risk of volume instability. The water requirement and the initial setting time were increased for all cement paste samples, regardless of the age of the WBA used, while the temperature of the paste was lower in the case when samples were made with aged WBA which can be attributed to a decrease in free lime (and periclase) after one year of storage. The final setting time increased for all paste samples tested with aged WBA. Nevertheless, the heat of hydration per g of cement was still higher, indicating reactivity of the WBA even after one year of storage in the closed containers.

Under open storage conditions (simulating an open landfill), the predominant mechanism was hydration. Ashes with higher CaO content reacted more rapidly with moisture in the air. There was also significant carbonation, after only 3 months. It was expected that these phase changes of the material would also affect the properties of composites where WBA is used. Based on the result, it is not recommended to store reactive WBA in air, to avoid loss of its reactivity.

The comparison between the two storage conditions clearly showed that the WBA samples stored in air exhibited faster aging than those stored in closed containers. Based on the results obtained, it is recommended that WBA be collected and stored immediately in closed containers to prevent pre-hydration and carbonation.

This research should facilitate the assessment of applicability by end users (power plants as WBA producers and concrete producers as WBA users). The current research is valuable to power plants and concrete producers as it provides recommendations for the storage of WBA. Proper storage of WBA can ensure its use in cementitious composites and minimise the negative effects of improper storage on the beneficial cementitious properties of WBA.

## Figures and Tables

**Table 1:** Chemical and physical properties of the wood biomass ash (WBA) samples and cement.

Parametar	CEM I	WBA1	WBA2	WBA3	WBA4	WBA5
P_2_O_5_ (mass %)	0.22	2.60	1.84	1.82	1.35	4.03
CaO (mass %)	59.80	48.70	51.90	46.75	16.25	47.35
MgO (mass %)	2.01	4.79	3.75	8.26	4.30	4.71
TiO_2_ (mass %)	0.23	0.15	0.15	0.34	1.17	0.25
SO_3_ (mass %)	3.33	4.77	3.58	2.73	0.60	3.95
Na_2_O_eq_ (mass %)	1.67	10.90	6.60	4.63	4.59	4.72
Pozzolanic oxides (SiO_2_+Al_2_O_3_+Fe_2_O_3_)	29.97	12.29	13.03	28.81	54.68	19.70
Free CaO (mass %)	2.50	8.60	13.50	7.30	0.50	8.80
Free MgO (mass %)	0.75	4.20	3.80	3.30	0.50	4.50
Cl^-^ (mass %)	0.04	0.06	<0.003	0.04	0.04	<0.003
LOI (950 °C)	3.6	13.4	13.8	3.8	8.3	12.7
pH	12.86	13.37	13.25	13.15	12.97	13.22
Density (g/cm^3^)	3.1	2.59	2.59	2.59	2.63	2.33
Bulk density (kg/m^3^)	n/a	0.38	0.38	0.91	0.61	0.55
d_50_ (μm)	9.6	18.2	43.3	71.9	120.7	17.8
Technology used	-	Pulverized fuel combustor	Pulverized fuel combustor	Grate combustor	Grate combustor	Bubbling fluidized bed
Type of wood used	-	Beech, oak, hornbeam, poplar, cherry	Beech, oak, hornbeam	Beech, oak, abies, picea	Beech, oak, hornbeam	Beech, oak, hornbeam, poplar
Average temperature, °C	-	700–750	700–750	700–950	800	850

## Data Availability

The data presented in this study are available in Supplementary Materials.

## Supplementary Materials

The following are available online at https://www.mdpi.com/article/10.3390/ma14071632/s1: Excel File.
